# Silent Inflammation: A Case of Blurry Vision Raising Diagnostic Challenges in an Elderly Patient

**DOI:** 10.7759/cureus.74822

**Published:** 2024-11-30

**Authors:** Meghan Beard, Emily Huang, Shariq Hashmi, Saika Sharmeen

**Affiliations:** 1 Department of Medicine, Division of Rheumatology, Allergy and Immunology, Stony Brook University Hospital, Stony Brook, USA; 2 Department of Pathology, Stony Brook University Hospital, Stony Brook, USA; 3 Department of Ophthalmology, Stony Brook University Hospital, Stony Brook, USA

**Keywords:** arteritic anterior ischemic optic neuropathy, giant cell arteritis (gca), normal esr, sudden vision loss, temporal artery biopsy (tabx), temporal artery ultrasound (taus)

## Abstract

Giant cell arteritis (GCA) is a large vessel vasculitis with cranial and extracranial vessel involvement. Clinicians suspect GCA when a patient exhibits symptoms or exam findings of temporal headache with sudden vision loss, jaw or tongue claudication, scalp tenderness, abnormal temporal artery exam, and diagnostic findings, including elevated inflammatory markers. We present a case, which highlights that, despite established diagnostic measures, challenges persist. The unexpected presentation serves as a valuable reminder of the condition’s diverse manifestations.

## Introduction

Giant cell arteritis (GCA) is one of the known vasculitides affecting large and medium-sized arteries. It most commonly affects the aorta, branches of the ophthalmic artery, and extracranial branches of the carotid artery [1]. GCA typically affects individuals over the age of 50, with the average age in the 2000s being 79 [2]. Incidence and prevalence have been shown to increase with age. People originating from Scandinavia have had the highest incidence and have been thought to be related to northern latitude locations. Women are more commonly affected than men with a ratio of 2.5:1. GCA has been shown to have a relation to polymyalgia rheumatica (PMR). The criteria for GCA hold point value for symptoms or exam findings such as morning stiffness in the shoulders/neck, sudden vision loss, jaw or tongue claudication, new temporal headache, scalp tenderness, abnormal temporal artery exam, and diagnostic findings, including erythrocyte sedimentation rate (ESR) > 50 mm/hour, or C-reactive protein (CRP) >10 mg/L, positive temporal artery biopsy or halo sign on ultrasound, bilateral artery involvement, and fluorodeoxyglucose positron emission tomography (FDG-PET) activity throughout the aorta [3]. Using these criteria, it has been found to have 87% sensitivity and 94.8% sensitivity. Biopsy-proven GCA has a 100% sensitivity and 94.9% specificity.

A temporal artery biopsy remains the gold standard for diagnosis given it has a specificity of 100%. Sensitivity varies widely from 50-80%. False-negative results are reported to be between 5% and 9% due to the presence of skip lesions [4,5]. Due to its invasive nature, the procedure carries a small risk of scalp necrosis, facial nerve injury, and stroke, though it is still considered a low-risk procedure. Pathology generally reflects transmural inflammatory infiltrate consisting of lymphocytes and macrophages. Giant cells are seen in about 75% of cases. Granulomatous inflammation is often seen in the arterial wall surrounding the internal elastic lamina. Inflammatory cell infiltration and proliferation of endothelial cells cause thickening of the intima. The overall inflammatory process causes fragmentation of the internal elastic lamina. In severe cases, there may be fibrinoid necrosis, though other etiology should be excluded [6].

This case highlights that, despite the previously mentioned measures for diagnosing GCA, there remain challenges. The variation from what is expected is a useful reminder of the many possible presentations.

## Case presentation

A 78-year-old male, with a past medical history of right eye amblyopia, hypertension, hyperlipidemia, and cataracts repaired in 2018, presented with right eye blurred vision. The patient initially had foggy vision in his right eye four days prior to admission. He was evaluated by an ophthalmologist at that time and noted to have a visual acuity of 20/60 in the right eye and 20/20 in the left eye. Two days later, he was seen by a retinal specialist who noted a decrease in vision to 20/150 in the right eye, and the left remained at 20/20. He was diagnosed with nonarteritic anterior ischemic optic neuropathy with accompanying swollen optic nerve at that time.

Within the next three days, he noted a significant deterioration in vision and was only able to discern shapes and outlines. He noted one episode of mild headache within this time, which resolved with acetaminophen and did not recur. The remaining review of systems was negative. A follow-up with his outpatient ophthalmologist revealed worsening vision, with bilateral optic nerve swelling, flame, and intraretinal edema, along with macular whitening. His right eye showed nonreactivity, and the left eye had a sluggish response to light prompting presentation to the hospital.

Upon presentation, the patient was hypertensive, with a blood pressure of 161/85 mmHg, with other vitals normal; heart rate of 73 beats per minute; temperature of 37 degrees Celsius; and oxygen saturation of 97% on room air. Physical exam was pertinent for non-tender temporal areas and slightly diminished temporal pulsation on the right side, without bruits appreciated. There was no temporal engorgement. A neurologic exam was without a focal deficit. Strong distal pulses were noted. Laboratory evaluation at the time of admission was significant for mild normocytic anemia (12.8 g/dL) (reference range: 13.2-17.8 g/dL). The ESR was mildly elevated at 36 mm/hour (reference range: 0-30 mm/hour), and CRP was normal at 0.4 mg/dL (0-0.5 mg/dL). Magnetic resonance imaging (MRI) brain and orbits with and without gadolinium were without significant abnormality, and magnetic resonance angiography (MRA) head and neck were without significant stenosis.

Due to concern for possible GCA, the patient was started on 1 g of intravenous (IV) methylprednisolone upon presentation given his rapid progression of vision loss, which was changed to 250 mg IV methylprednisolone every six hours the following day. The ESR decreased the following day from 36 mm/hour to 32 mm/hour (reference range: 0-30 mm/hour) and CRP decreased from 0.4 mg/dL to 0.3 mg/dL (0-0.5 mg/dL). A temporal artery ultrasound was done, which showed mild intimal thickening bilaterally without significant stenosis or flow disturbance. The duplex of the bilateral axillary arteries showed no evidence of stenosis or flow disturbance. Bilateral axillary arteries were patent with normal triphasic waveform. Laboratory evaluation was negative for protein C, protein S, lupus anticoagulant, anticardiolipin, beta-2-glycoprotein IgM and IgG, double-stranded DNA, and anti-neutrophil cytoplasm antibodies (ANCA). Infectious workup was negative for Ebstein-Barr virus, herpes simplex virus, malaria, babesia, Lyme disease, syphilis, COVID-19, or parasite smear.

**Figure 1:**
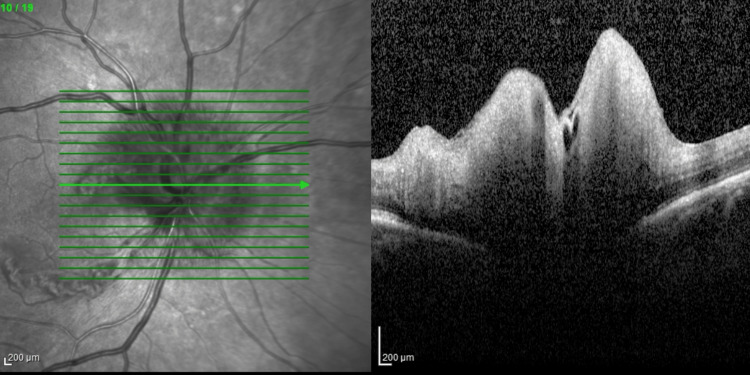
Optical coherence tomography (OCT) image of the right optic nerve. The en-face image is seen on the right, which shows the blurring of the optic disc margin. A cross-section through the center of the nerve is shown on the left, in which elevation of the optic nerve head is visible. These findings together indicate that the right optic nerve head is edematous.

**Figure 2:**
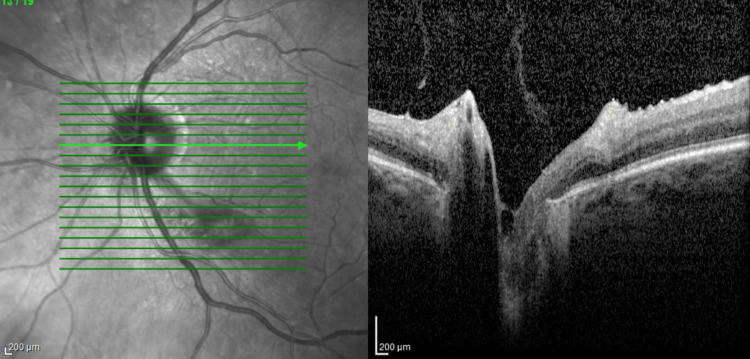
OCT image of the left optic nerve. The en-face image shows more clear margins of the optic nerve. The cross-section of the optic nerve head similarly does not show clear elevation of the optic nerve.

**Figure 3:**
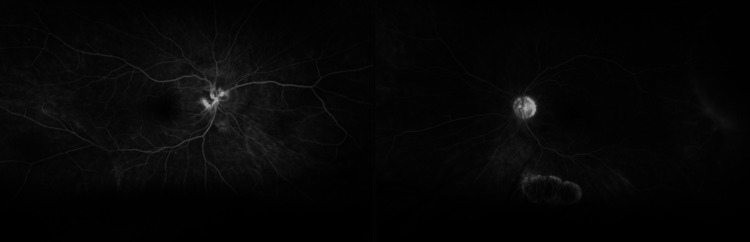
Fluorescein angiogram of both eyes. This is a late, recirculation phase frame of the fluorescein angiogram of both eyes. The left eye (on the left) shows leakage at the optic disc, which may occur in optic disc edema. The right eye has a chorioretinal scar inferior to the macula. Some decreased staining is seen in the temporal periphery, indicating a possible region of retinoschesis.

He did not have improvement in vision in the following days. He continued to deny fever, chills, joint pain, jaw claudication, and headaches. He refused the ophthalmic exam the day following admission. Two days later, his visual acuity worsened to light perception in the right eye and hand motion in the left eye. A dilated fundus exam showed grade three disc edema bilaterally (Figures 1-3).

**Figure 4:**
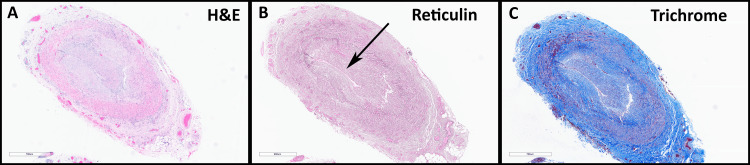
Temporal artery biopsy. A. H&E sections show scattered foci of chronic inflammatory cells, including histiocytes, lymphocytes, and rare giant cells, involving the arterial wall. Inflammatory infiltrates involve the intima, media, and the inner adventitia. B. Reticulin stains performed show focal fragmentation of the elastic layer. C. Trichrome stains highlight areas of intimal fibrosis. All images are at a total magnification of 40x.

A bilateral temporal artery biopsy was performed three days later. H&E sections showed scattered foci of chronic inflammatory cells, including histiocytes, lymphocytes, and rare giant cells, involving the arterial wall. Inflammatory infiltrates involve the intima, media, and inner adventitia. Reticulum stains performed showed focal fragmentation of the elastic layer (Figure 4). Trichrome stains highlighted areas of intimal fibrosis. Visual acuity again worsened to no light perception in the right eye and light perception in the left eye.

The patient was continued on IV methylprednisolone for five days, then transitioned to 80 mg oral prednisone daily. Interleukin-6 was normal, and hepatitis and QuantiFERON testing were negative. The patient was discharged from the hospital with a script for a computed tomography angiography (CTA) chest, abdomen, and pelvis with and without IV contrast to evaluate for extracranial large vessel involvement. The CTA did not show evidence of vasculitis. Throughout the course, his vision never improved. Unfortunately, he was thereafter lost to follow-up.

## Discussion

The primary differentials in this case would be based on the ophthalmic exam. The sudden painless asymptomatic vision loss may have a large differential, but given the patient’s age and eye exam, it was narrowed down to two main differentials: arteritic vs. non-arteritic anterior ischemic optic neuropathy (AION). AION is caused by ischemia of the optic nerve involving the most anterior 1 mm portion of the optic nerve, which is known as the optic disc. The optic disc can be viewed on retina examination and can be seen as swollen in AION. There are two types of AION: arteritic AION (AAION) and nonarteritic AION (NAION). AAION is almost always due to GCA. It initially presents as unilateral but rapidly becomes bilateral. NAION pathophysiology is most often idiopathic. Possible associations with NAION have been hypertension, hyperlipidemia, diabetes mellitus, and obstructive sleep apnea. NAION is due to a lack of blood flow to the optic nerve, usually related to microvascular risk factors, and does not involve arteritis. Thus, a temporal artery biopsy in patients with NAION would appear normal [7]. Our patient’s temporal artery biopsy showed inflammatory infiltrates and fragmentation of the internal elastic lamina, suggesting GCA [8] (Figure 4). The rapid progression to vision loss, coupled with the temporal artery biopsy findings in our patient, supports a diagnosis of GCA.

When suspecting GCA, the initial workup should include the evaluation of inflammatory markers, including ESR-CRP and platelets. CRP has been found to be a more sensitive marker than ESR in patients with temporal artery biopsy-proven GCA, though both ESR and CRP being elevated has shown even greater odds [9]. About 4% of patients have been shown to have normal ESR-CRP at the time of diagnosis. These markers are nonspecific, and other additional causes should be ruled out accordingly (infection, malignancy, etc.).

The accuracy of ultrasound (US) in the diagnosis of GCA has been controversial in recent times; however, it remains an important aspect of diagnostic criteria. Some of the benefits of US use include lack of radiation exposure, non-invasiveness, cost, and availability. Controversy centers around the skill and technique of the sonographer. US visualizes the temporal artery wall and lumen to determine flow characteristics. To decrease operator variability, a compression sign was developed, which is positive if the temporal artery is visible while compressing the artery. This was found to have 79% sensitivity and 100% specificity. The distinct “halo sign” is described as a hypoechogenic perivascular stricture. It is thought to be due to wall edema caused by inflammation [10]. Our patient interestingly had normal bilateral temporal artery US. Given the atypical presentation in our patient, we also performed bilateral axillary US. In a recent study, the sensitivity of sonographic imaging for the final diagnosis of GCA increased from 69.6% to 84.8%, when axillary US results were considered in addition to temporal artery US, while the specificity remained high [11]. Unfortunately, both temporal and axillary US were nonrevealing in our patient.

More recently, FDG PET-CT has been utilized for extracranial manifestations of GCA such as aortitis. About 50% of patients with GCA have been estimated to also have aortitis [12]. While it is not recommended for the evaluation of cranial arteries, research is ongoing for its utility. Imaging must be performed within 72 hours of starting steroids as the hypermetabolism signal decreases thereafter [13]. A retrospective study found FDG PET/CT use for detecting hypermetabolism in the cranial arteries was 64-82% sensitive and 100% specific.

Similar cases of GCA with normal inflammatory markers have been reported in the literature [14-18]. Martins et al. [15] described a case report of a patient with biopsy and US-proven GCA, with arteritic AION, which eventually also led to vision loss; however, their patient presented with a headache. They also identified 31 case reports and small case series, describing a total of 41 patients with the diagnosis of GCA and low or normal inflammatory markers. There was a predominance of female individuals (63%). The mean age is 69.4 ± 12.5 years, with headache and eye involvement being the most frequent clinical manifestations reported (81% and 59%, respectively). Diplopia and visual loss were the most common eye symptoms described, and 14/24 (58%) patients with visual involvement were reported to have visual loss. Less than one-quarter of the patients complained of jaw claudication, polymyalgia rheumatica, or constitutional symptoms [15]. Mahgoub et al. reported a case of GCA with normal inflammatory markers, but the patient presented with constitutional symptoms and proximal myopathy. GCA was suspected based on a physical exam, suggesting decreased temporal artery pulses [14]. Rebello et al. [16] reported a more recent classic case of GCA wherein a 70-year-old male presented with a three-week history of headache and jaw claudication with normal ESR and CRP; however, he did not have any visual disturbance, as seen in our patient. Singh et al. [18] discussed a very similar case to the one presented here; however, their patient presented with a headache, and initiation of steroid treatment led to resolution of symptoms. Our patient’s case was difficult as he did not have any of the classic GCA symptoms and he had risk factors for NAION.

This case highlights the complexities in diagnosing GCA. The rapid progression of vision loss in our patient, coupled with his age and clinical presentation, raised significant concern for GCA, despite near-normal inflammatory markers. The initial underappreciation for the severity of his condition illustrates the diagnostic challenges faced by clinicians.

The decision to initiate high-dose intravenous corticosteroids was crucial given the risk of GCA-related vision loss. Although the patient demonstrated some reduction in inflammatory markers following steroid therapy, there was no corresponding improvement in visual acuity. This emphasizes the need for timely intervention in suspected GCA cases, as delayed treatment can lead to irreversible vision loss. The temporal artery biopsy provided definitive evidence of GCA, reinforcing the importance of this diagnostic tool in cases with atypical presentations.

## Conclusions

This case highlights a unique presentation of GCA. This serves as a reminder to avoid anchoring a diagnosis and complete diagnostic evaluation with a broad differential. Though uncommon, atypical presentations are possible, including a lack of symptoms and normal inflammatory markers. In addition to normal inflammatory markers, this patient additionally had an unremarkable ultrasound, prompting the need for a temporal artery biopsy. While a temporal artery biopsy is not always necessary to meet the criteria for GCA, it was required in this case for final confirmation. The rapid progression of vision loss, despite early pulse dose steroid initiation, underscores the importance and urgency of recognizing the disease, reminding clinicians to begin treatment early.
